# Prediction and classification of aminoacyl tRNA synthetases using PROSITE domains

**DOI:** 10.1186/1471-2164-11-507

**Published:** 2010-09-22

**Authors:** Bharat Panwar, Gajendra PS Raghava

**Affiliations:** 1 Bioinformatics Centre, Institute of Microbial Technology, Chandigarh, India

## Abstract

**Background:**

Aminoacyl tRNA synthetases (aaRSs) catalyse the first step of protein synthesis in all organisms. They are responsible for the precise attachment of amino acids to their cognate transfer RNAs. There are twenty different types of aaRSs, unique for each amino acid. These aaRSs have been divided into two classes, each comprising ten enzymes. It is important to predict and classify aaRSs in order to understand protein synthesis.

**Results:**

In this study, all models were developed on a non-redundant dataset containing 117 aaRSs and an equal number of non-aaRSs, in which no two sequences have more than 30% similarity. First, we applied the similarity search technique, BLAST, and achieved a maximum accuracy of 67.52%. We observed that 62% of tRNA synthetases contain one or more domains from amongst the following four PROSITE domains: PS50862, PS00178, PS50860 and PS50861. An SVM-based model was developed to discriminate between aaRSs, and non-aaRSs, and achieved a maximum MCC of 0.68 with accuracy of 83.73%, using selective dipeptide composition. We developed a hybrid approach and achieved a maximum MCC of 0.72 with accuracy of 85.49%, where SVM model developed using selected dipeptide composition and information of four PROSITE domains. We further developed an SVM-based model for classifying the aaRSs into class-1 and class-2, using selective dipeptide composition and achieved an MCC of 0.79. We also observed that two domains (PS00178, PS50889) in class-1 and three domains (PS50862, PS50860, PS50861) in class-2 were preferred. A hybrid method was developed using these domains as descriptor, along with selected dipeptide composition, and achieved an MCC of 0.87 with a sensitivity of 94.55% and an accuracy of 93.19%. All models were evaluated using a five-fold cross-validation technique.

**Conclusions:**

We have analyzed protein sequences of aaRSs (class-1 and class-2) and non-aaRSs and identified interesting patterns. The high accuracy achieved by our SVM models using selected dipeptide composition demonstrates that certain types of dipeptide are preferred in aaRSs. We were able to identify PROSITE domains that are preferred in aaRSs and their classes, providing interesting insights into tRNA synthetases. The method developed in this study will be useful for researchers studying aaRS enzymes and tRNA biology. The web-server based on the above study, is available at http://www.imtech.res.in/raghava/icaars/.

## Background

Aminoacyl tRNA synthetases (aaRSs) play a central role in protein translation by covalently linking the correct amino acid to its cognate transfer RNA [1]. This covalent linkage is a two-step aminoacylation reaction and ensures the fidelity of translation of the genetic code. In the first step, an amino acid (aa) activated by ATP, releases pyrophosphate (PPi) and is converted into the aminoacyl-adenylate (aa-AMP) complex. This complex remains bound to the tRNA synthetase. In the second step, the activated amino acid is transferred onto the 2'-terminal or 3'-terminal ribose of the corresponding tRNA (aa-tRNA). The aaRSs also perform editing activity by clearance of mischarged tRNA [2]. The editing activity is shown by both class-1 (ValRS, IleRS and LeuRS) and class-2 (ThrRS, AlaRS, ProRS and PheRS) tRNA synthetases [3]. The defects in editing activity of aaRSs can be lethal and may lead to many pathological problems e.g. neuronal pathologies (encephalopathy, cerebellar ataxia and peripheral neuropathy), autoimmune disorders and disrupted metabolic conditions [4-8].

Studies of tRNA and tRNA synthetases from bacteria, fungi, plants and mammals have shown that there are twenty aminoacyl tRNA synthetases in all organisms and each is specific for a single amino acid [9]. Aminoacyl-tRNA synthetases differ in amino acid sequence length, three-dimensional structure, molecular weight, and subunit organization, and have limited sequence homology [10-12]. Based on the multiple sequence analysis and the architecture of catalytic sites, aaRSs are divided into two classes of ten aaRSs each [13]. The structural characteristics of the catalytic domains of all aaRSs reveal that the active sites of class-1 enzymes contain the classical Rossmann dinucleotide-binding fold and two signature peptides, HIGH and KMSKS. The active sites of class-2 aaRSs contain an anti-parallel β-sheet flanked by helices on both sides, and have three (motif 1, motif 2 and motif 3) signature motifs [14,15]. The catalytic site-based partition of aaRSs into two classes provides a strong correlation with function. An amino acid is transferred onto the 2'-OH group of the ribose of last nucleotide of tRNA by class-1 (ArgRS, CysRS, GlnRS, IleRS, LeuRS, GluRS, MetRS, TrpRS, TyrRS & ValRS) and the 3'-OH group by class-2 (AlaRS, AsnRS, AspRS, GlyRS, HisRS, LysRS, PheRS, ProRS, SerRS & ThrRS) tRNA synthetases [13]. Two synthetases PheRSs and LysRSs are exceptions to this rule. All known PheRSs belong to class-2, going by their structural characteristics, but transfer amino acids onto the 2'-OH group [16,17]. The lysyl-tRNA synthetases are found to belong both class-1 and class-2. Most of the LysRSs are found in class-2 in many eubacteria and all eukaryotes, while class-1 LysRSs are mainly found in archea and some eubacteria [18,19]. This class-determination is very important for the functional annotation of tRNA synthetases. At the present time, genome sequencing projects continuously produce huge amounts of sequence data, but the function of several proteins is still unclear. Some aaRSs have been recognized as validated drug targets and one representing a potential drug target has been identified from each of the essential twenty aaRSs [20]. Earlier many methods have been developed for the prediction of DNA or RNA binding proteins [21-25] and further classification into mRNA, rRNA, tRNA & snRNA binding protein [21,22]. In this paper, an attempt has been made to predict and classify aaRSs from the primary structure of protein. A novel hybrid approach based on SVM and PROSITE has been adopted in order to predict the aaRSs and further classify them into two classes. In this hybrid approach, most distinguishable domains were selected from the PROSITE database by using the ProfileScan method of InterProScan, and integrated in composition-based SVM models. The domains PS50862, PS00178, PS50860 and PS50861 were used for the prediction of aaRSs and one additional domain PS50889 was integrated with SVM for the classification of aaRSs into class-1 and class-2.

## Results

### Sequence Similarity Search

It is a common trend to annotate a protein sequence using similarity-based approach like BLAST. Thus, we developed a BLAST- based approach for discriminating aaRSs and non-aaRSs. As shown in Table 1, we achieved a maximum of 67.52% accuracy at 10E-value. We have used the same strategy for the BLAST-based discrimination between class-1 and class-2 aaRSs and found that the accuracy of BLAST at 10E-value was 72.77%. It is possible to develop methods that result in higher accuracy with respect to BLAST-like method. Thus, there is a need to develop models based on machine learning techniques to discriminate aaRSs versus non-aaRSs with high accuracy.

**Table 1:** The performance of BLAST at E-value threshold 10.

**S. No**.	Method	Sensitivity	Specificity	Accuracy*
1	Discrimination between aaRSs and Non-aaRSs	74.36	60.68	67.52

2	Discrimination between class-1 and class-2 aaRSs	71.7	73.85	72.77

* The number of aaRSs/class-1 and non-aaRSs/class-2 having no hit (target) were considered as false negative and false positive respectively.

We have developed two different types of prediction methods: (1) prediction of aaRSs, and (2) discrimination between class-1 and class-2 aaRSs. For each prediction, we applied five approaches: (i) domain-based approach, (ii) SVM modules using amino acid composition, (iii) SVM modules using dipeptide composition, (iv) Hybrid approach^1 ^based on SVM using composition and PROSITE domains and (v) Hybrid approach^2 ^based on SVM using dipeptide composition and PROSITE domains. We trained and tested all our models on a 30% non-redundant dataset of aaRSs, non-aaRSs, class-1 and class-2 aaRSs.

### (1) Prediction of aminoacyl tRNA Synthetases

First we developed prediction tools for discriminating between aaRSs and non-aaRS. We used aaRSs and non-aaRSs as positive and negative instances respectively.

#### (i) Domain-based approach

The weight matrix based signature profile gives evolutionary information of any protein family or group of protein sequences. It is possible to discriminate some protein families based on the distinguishable constant and variable regions [26]. So we used PROSITE from the ProfileScan method of InterProScan [27]. We used aaRSs and non-aaRSs protein sequences, each group represented by 117 sequences. We analysed all profiles and selected the four most distinguishable domains which were PS50862, PS00178, PS50860 and PS50861. The accession numbers of ProfileScan and the short names of these domains are AA_TRNA_LIGASE_II, AA_TRNA_LIGASE_I, AA_TRNA_LIGASE_II_ALA and AA_TRNA_LIGASE_II_GLYAB respectively. It has been observed that 62% of aaRSs contain at least one of these PROSITE domains; about 9.4% of non-aaRSs also contain one of the above-mentioned domains. This indicates that a domain based approach is not sufficient for discriminating amongst all aaRSs. Thus, there is a need to develop sophisticated techniques, in combination with domain-based approaches. An SVM-based classifier has been developed, using four dimensions of vector, one for each domain; this achieved 61.41% sensitivity, 90.65% specificity, 76.08% accuracy and 0.54 MCC. In this dataset ~38% tRNA synthetases lacked any distinguishable PROSITE domains.

#### (ii) SVM modules using amino acid composition

**Figure 1:**
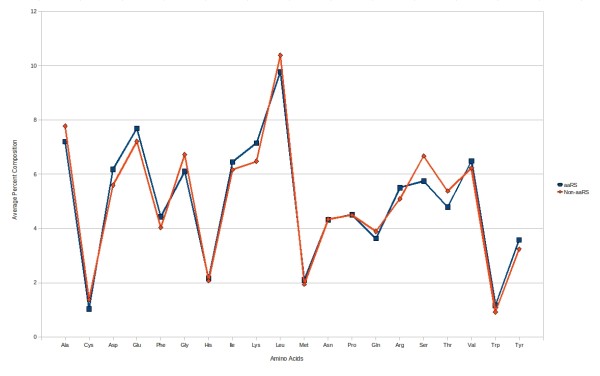
**Percent composition of each of 20 amino acids in aaRS and non-aaRS proteins**.

It has been shown in the past that amino acid composition can be used to classify the different classes of proteins and developments of prediction tools using machine learning techniques [28,29]. We analysed amino acid compositions of aaRS and non-aaRS proteins (Figure 1). It has been observed that amino acid composition of aaRS and non-aaRS protein was significantly different from each other. Thus, it was possible to discriminate aaRS proteins from other proteins based on amino acid composition. The SVM-based classifier has been developed using 20 dimensions of vector, one for each amino acid. Different kernels and parameters of SVM have been tried and optimized for the best discrimination between aaRSs and non-aaRSs. We achieved 74.38% sensitivity, 82.97% specificity, 78.63% accuracy and 0.60 MCC.

#### (iii) SVM modules using dipeptide composition

**Figure 2:**
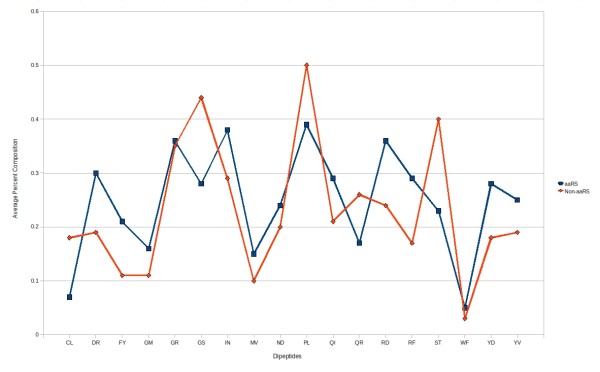
**Percent composition of selected 18 dipeptides in aaRS and non-aaRS proteins**.

In the previous studies, it has been shown that dipeptide composition-based methods are more successful than amino acid composition-based methods in the classification of proteins [30], because the former incorporate the fraction of amino acids as well as their local order (which make them more informative than amino acid composition). An SVM-based classifier was developed using 400 dimensions of vector (20 × 20) of dipeptide compositions, one for each dipeptide and this achieved 93.99% sensitivity, 62.43% specificity, 78.20% accuracy and 0.60 MCC. We used SVM attribute selection method by using WEKA and selected 18 distinguishable dipeptides (CL, DR, FY, GM, GR, GS, IN, MV, ND, PL, QI, QR, RD, RF, ST, WF, YD and YV). The compositions of these 18 dipeptides were significantly different in aaRS and non-aaRS protein sequences (figure 2). Different kernels and parameters of SVM were tried, optimized and achieved maximum 87.14% sensitivity, 80.15% specificity, 83.74% accuracy and 0.68 MCC.

#### (iv) Hybrid approach^1 ^based on SVM using Composition and PROSITE domains

In this hybrid approach^1^, we combined the 20 features of amino acid composition and 4 features of selected four domains of PROSITE and generated a vector of 24 dimensions. We developed SVM based model and achieved 80.36% sensitivity, 79.64% specificity, 79.93% accuracy and 0.60 MCC.

#### (v) Hybrid approach^2 ^based on SVM using Dipeptide and PROSITE domains

**Figure 3:**
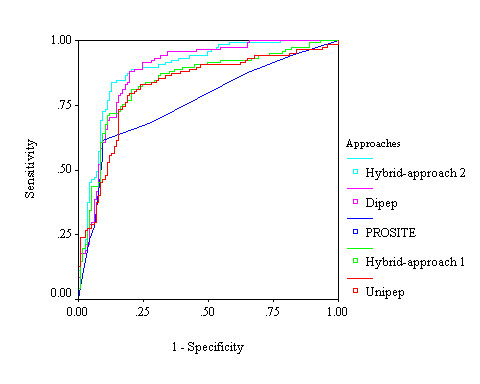
**Performance of aminoacyl tRNA synthetases prediction of different (amino acid composition, dipeptide composition, domain based hybrid approach^1^, and hybrid approach^2^) SVM modules in a threshold-independent manner by receiver operating characteristic plot**.

We generated a vector of 404 dimensions, which contain 400 features of dipeptide composition and 4 features of the selected four domains of PROSITE. We applied SVM based learning using this vector and achieved 84.53% sensitivity, 70.91% specificity, 77.76% accuracy and 0.57 MCC. In the *hybrid approach*^2^*_, _*we generated a vector of 22 dimensions, using composition of 18 selected dipeptides and 4 features of PROSITE domains. We developed SVM model using above vector of dimensions 22 and achieved 83.73% sensitivity, 87.10% specificity, 85.49% accuracy and 0.72 MCC. We observed that hybrid approach^2 ^performed much better than any other approaches developed in this study for predicting aaRSs (figure 3 and table 2).

**Table 2:** The performance of SVM modules developed for discriminating aaRSs and non-aaRSs.

**S. No**.	Name of Approach	Sensitivity	Specificity	Accuracy	MCC
1	Domain-based model	61.41	90.65	76.08	0.54

2	Amino acid Composition	74.38	82.97	78.63	0.6

3	Dipeptide composition	87.14	80.15	83.74	0.68

4	Hybrid approach^1^*	80.36	79.64	79.93	0.6

5	Hybrid approach^2^**	83.73	87.1	85.49	0.72

* Hybrid approach^1 ^SVM model based on amino acid composition domain features

**Hybrid approach^2 ^SVM model based on selected dipeptide composition and domain features

### (2) Discriminating class-1 and class-2 aaRSs

We further developed tools for discriminating class-1 and class-2 aaRSs. We used class-1 and class-2 aaRSs as positive and negative instances respectively. We applied same approaches and techniques, which have been used for the prediction of aaRSs.

#### (i) Domain-based approach

We analysed all protein sequences of class-1 and class-2 aaRSs by using ProfileScan. We selected five most distinguishable domains, which were PS50862, PS00178, PS50860, PS50861 and PS50889 (accession number of ProfileScan). Domains PS50862, PS00178, PS50860 and PS50861 have been already used for the prediction of aaRSs. One additional domain PS50889 is an S4 RNA-binding domain profile and commonly found in tyrosyl-tRNA synthetases (class-1). It was observed that two domains (PS00178, PS50889) in class-1 and three domains (PS50862, PS50860, PS50861) in class-2 are preferred. A SVM-based classifier was developed using 5 features, one for each domain, and achieved 100.00% sensitivity, 53.85% specificity, 74.64% accuracy and 0.56 MCC. In this case, class-1 was considered as positive and class-2 as negative instances. It is shown in the results that the method was able to predict class-1 with 100% accuracy but failed to predict class-2. Thus there is a need to develop a method which can discriminate two classes with reasonable accuracy.

#### (ii) SVM modules using amino acid composition

**Figure 4:**
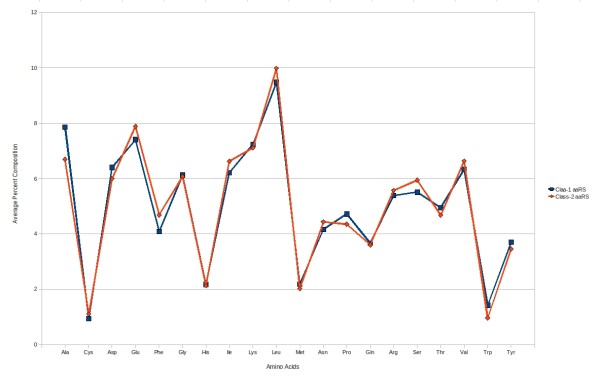
**Percent composition of each of 20 amino acids in class-1 aaRS and class-2 aaRS proteins**.

We analysed and compared amino acid composition of class-1 and class-2 aaRS proteins, and observed significant differences between amino acid compositions (Figure 4). We applied SVM using amino acid composition for discriminating two classes and achieved 68.00% sensitivity, 64.62% specificity, 66.09% accuracy and 0.33 MCC.

#### (iii) SVM modules using dipeptide composition

**Figure 5:**
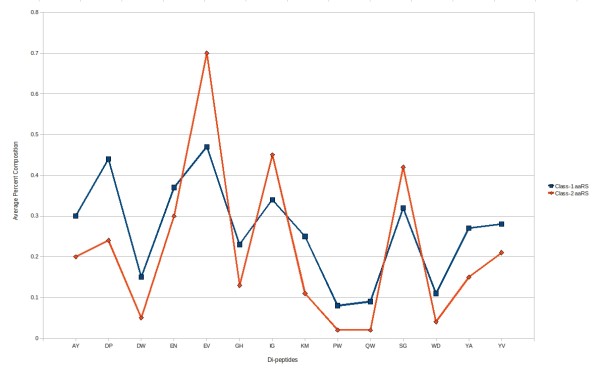
**Percent composition of selected 14 dipeptides in class-1 aaRS and class-2 aaRS proteins**.

An SVM classifier has been developed using a vector of 400 dimensions (dipeptide composition) and it achieved 88.55% sensitivity, 61.54% specificity, 73.51% accuracy and 0.52 MCC. We used SVM attribute selection method by using WEKA and selected 14 distinguishable dipeptides (AY, DP, DW, EN, EV, GH, IG, KM, PW, QW, SG, WD, YA and YV). The composition of these 14 dipeptides was significantly different in class-1 and class-2 aaRS protein sequences (figure 5). Different kernels and parameters of SVM were tried, optimized and achieved 85.27% sensitivity, 92.31% specificity, 88.99% accuracy and 0.79 MCC.

#### (iv) Hybrid approach^1 ^based on SVM using composition and PROSITE domain

In the hybrid approach^1^, a vector of 25 dimensions was created from five domains and the 20 features of amino acid composition. Finally, an SVM-based model has been developed using the above vector and this achieved 81.09% sensitivity, 83.08% specificity, 82.18% accuracy and 0.65 MCC.

#### (v) Hybrid approach based on SVM using Dipeptide and PROSITE domain

**Figure 6:**
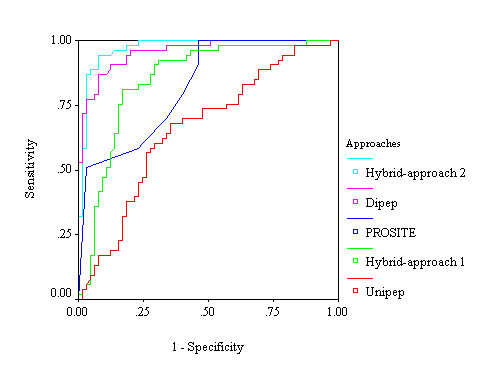
**Performance of discrimination between class-1 and class-2 aaRSs of different (amino acid composition, dipeptide composition, domain based hybrid approach^1^, and hybrid approach^2^) SVM modules in a threshold-independent manner by receiver operating characteristic (ROC) plot**.

We generated a total of 405 dimensions of vector by using 400 features of dipeptide composition and 5 features of the selected five domains of PROSITE. We applied SVM-based learning and achieved 86.55% sensitivity, 80.00% specificity, 82.94% accuracy and 0.68 MCC. In the *hybrid approach^2^_, _*we generated a total of 19 dimensions of vector by using 14 features of selected dipeptides and 5 features of PROSITE domains. We achieved maximum 94.55% sensitivity, 92.31% specificity, 93.19% accuracy and 0.87 MCC. Here also we observed that SVM-based hybrid approaches^2 ^performed much better than other four approaches for the discrimination between class-1 and class-2 aaRSs (figure 6 and table 3).

**Table 3:** The performance of SVM modules developed for discriminating class-1 and class-2 aaRSs.

**S. No**.	Name of Approach	Sensitivity	Specificity	Accuracy	MCC
1	Domain-based model	100	53.85	74.64	0.56

2	Amino acid composition	68	64.62	66.09	0.33

3	Dipeptide composition	85.27	92.31	88.99	0.79

4	Hybrid approach^1^*	81.09	83.08	82.18	0.65

5	Hybrid approach^2^**	94.55	92.31	93.19	0.87

* Hybrid approach^1 ^SVM model based on amino acid composition domain features

** Hybrid approach^2 ^SVM model based on selected dipeptide composition and domain features

### Performance on the realistic dataset

**Figure 7:**
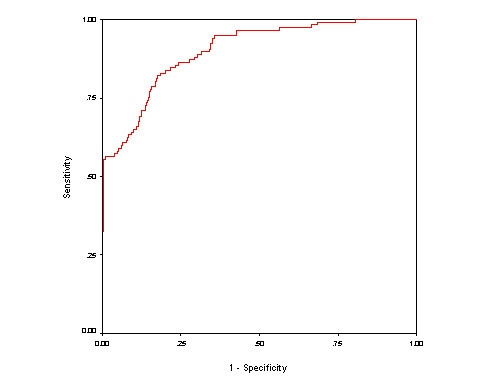
**The ROC plot of the SVM model performance (Hybrid approach^2^) on realistic dataset**.

In this study, we trained and tested our models on a dataset containing equal numbers of aaRSs and non-aaRSs. In reality, the number of aaRSs in a genome is not very high. Thus, we created a realistic dataset, which contains 117 aaRSs and 1200 non-aaRSs (nearly 1:10 ratio of aaRSs/positive and non-aaRSs/negative instances). We developed models using Hybrid approach^2 ^(selected dipeptide composition and domain features) for discriminating aaRSs and non-aaRSs on this realistic dataset. We achieved maximum 95.90% accuracy with 0.71 MCC (figure 7). These results demonstrate that our approach is also effective on realistic dataset.

### Performance on independent dataset

It is important to evaluate a newly developed model on an independent dataset, not used for its training or testing. In order to create independent dataset, we retrieved total 1293 newly added tRNA synthetases (08^th ^July 2009 to 18^th ^May 2010) from Swiss-Prot database. All the aaRSs having more than 30% sequence similarity with any other aaRSs have been removed. Thus, the final independent dataset contains 44 non-redundant aaRSs. We have further created negative datasets of 450 non-aaRSs. The performances of our model developed using Hybrid approach^2 ^have been evaluated at various thresholds. We observed the performance of hybrid approach^2 ^based SVM model and achieved maximum 95.75% accuracy with 0.71 MCC. Here also both the sensitivity and specificity shifted toward negative and positive thresholds respectively (table 4). The independent dataset contains 18 sequences of class-1 and 26 sequences of class-2 aaRSs. The Hybrid approach^2 ^based SVM model predicted 14 (77.78%) class-1 aaRSs and all 26 (100%) class-2 correctly.

**Table 4:** The performance of Hybrid approach^2 ^based SVM model on independent dataset.

Threshold	Sensitivity	Specificity	Accuracy	MCC
-1.0	100.00	43.11	48.18	0.25

-0.9	97.73	63.56	66.60	0.35

-0.8	81.82	82.44	82.39	0.43

-0.7	72.73	92.22	90.49	0.54

-0.6	63.64	97.11	94.13	0.63

-0.5	54.55	99.56	95.55	0.69

-0.4	52.27	100.00	95.75	0.71

-0.3	52.27	100.00	95.75	0.71

-0.2	52.27	100.00	95.75	0.71

-0.1	52.27	100.00	95.75	0.71

0.0	52.27	100.00	95.75	0.71

0.1	52.27	100.00	95.75	0.71

0.2	52.27	100.00	95.75	0.71

0.3	52.27	100.00	95.75	0.71

0.4	52.27	100.00	95.75	0.71

0.5	47.73	100.00	95.34	0.67

0.6	43.18	100.00	94.94	0.64

0.7	36.36	100.00	94.33	0.59

0.8	22.73	100.00	93.12	0.46

0.9	18.18	100.00	92.71	0.41

1.0	11.36	100.00	92.11	0.32

### Comparison with SVM-Prot

In the past, numbers of methods have been developed for predicting RNA-binding proteins and class of Enzymes (21-23). To the best of our knowledge, in this study, for the first time a method has been developed for prediction and classification of tRNA synthetases. SVM-Prot is one of the comprehensive methods developed for predicting enzyme families which includes tRNA synthetase. Thus, we evaluated the performance of SVM-Prot (22) on 117 aaRSs used in our main dataset. SVM-Prot predicts various possible enzyme classes for query protein, with probability score. We used the highest probability score (first hit of prediction) of SVM-Prot (table 5) for analyzing predictions. It was observed that only 13.68% of tRNA synthetases correctly predicted by SVM-Prot method. This demonstrates that our method is important for predicting and classifying aaRSs.

**Table 5:** Prediction results of SVM-Prot by using 117 aaRSs of main dataset.

**S.No**.	Predicted Result of SVM-Prot	Number of aaRSs
1	Zinc-binding	55

2	**EC 6.1.-.-: Ligases - Forming Carbon-Oxygen Bonds**	**13**

3	All DNA-binding	8

4	Iron-binding	8

5	EC 2.7.-.-: Transferases - Transferring Phosphorus-Containing Groups	5

6	Magnesium-binding	4

7	EC 3.6.-.-: Hydrolases - Acting on Acid Anhydrides	3

8	All lipid-binding proteins	3

9	Manganese-binding	3

10	Metal-binding	3

11	EC 3.1.-.-: Hydrolases - Acting on Ester Bonds	2

12	Aptamer-binding protein	1

13	EC 2.4.-.-: Transferases - Glycosyltransferases	1

14	EC 5.4.-.-: Isomerases - Intramolecular Transferases	1

15	**Ligases - Forming Carbon-Oxygen Bonds**	**1**

16	Photosystem II	1

17	**RNA-binding Proteins**	**1**

18	TC 3.A.5 Type II (general) secretory pathway (IISP) family	1

19	Transmembrane	1

20	**tRNA-binding Proteins**	**1**

21	Tyrosine Kinase Receptors	1

*Broad-level correctly predicted tRNA synthetase by SVM-Prot method is highlighted by bold font.

## Discussion

In recent years, rapid advances in genomics and proteomic studies have yielded a tremendous amount of data. The functional annotation of all these sequences using experimental approaches is a very labour-intensive and time-consuming process. Therefore, computational approaches are required to fill the gap. The prediction based functional annotation of all protein classes is not possible. Thus, it is important to concentrate on a single class of functionally important proteins. The aaRSs enzymes constitute a major protein class and play a vital role in protein synthesis. The catalytic activities of these aaRSs affect the determination of the genetic code. For this reason, they are essential for protein synthesis and cell viability. The catalytic specificity of aaRSs is necessary for cell survival. Many natural compounds and antibiotics specifically target aaRSs, and inhibit the growth or survival of the target bacteria. The aaRSs have been already recognized as validated drug targets. In the present scenario, drug-resistance is continuously increasing for existing antibiotics and we need more novel antimicrobial agents directed against the novel targets. From all essential twenty aaRSs, each one represents a potential drug target. But these are still poorly exploited drug targets and only one aaRS inhibitor mupirocin is a marketed drug, which is specifically targeted against the isoleucyl-tRNA synthetase [20]. The investigation of aaRS families by genomic and biochemical research has been suggested. In this respect, aaRSs constitute a promising platform for the development of novel-antibiotics, and these are predicted to have no cross-resistance to other classical antibiotics [31]. This protein family has limited sequence homology, therefore we need more powerful computational tools than BLAST and other similarity based methods.

In order to assist biologists in assigning the function of unknown aaRS proteins, a systematic attempt has been made for predicting aaRS proteins and their classes. We obtained aaRSs protein sequences from the ENZYME database of ExPASy and created class-1 and class-2 specific datasets. The creation of a negative dataset (non-aaRSs) is as important as positive datasets (aaRSs) for developing any classification method. Thus, we manually extracted non-aaRSs from Swiss-Prot by using appropriate searching options. We selected four most distinguishable PS50862, PS00178, PS50860 and PS50861 domains from PROSITE, which can discriminate aaRSs from non-aaRSs. In the classification between class-1 and class-2 aaRSs, we selected one more PS50889 (S4 RNA-binding) domain. The S4 is a small globular domain consisting of 60-65 amino acid residues, found in class-1 (tyrosyl-tRNA synthetases) and many other RNA-related protein families but absent in class-2 aaRSs. Domain PS00178 contains the 'HIGH' signature, which is a part of the adenylate binding site of class-1 aaRSs. Class-2 aaRSs do not share a high degree of similarity; however, at least three conserved regions are present and PS50862 is a domain of these conserved regions. PS50860 and PS50861 are the domains of AlaRS and GlyRS respectively and both belong to class-2 aaRSs. This information of domains was used for discrimination between class-1 (PS00178 and PS50889) and class-2 (PS50862, PS50860 and PS50861) aaRSs. We have also found domain PS50886, which is a signature of tRNA binding domain. This is widely distributed among different tRNA synthetases (class-1 and class-2 both) and found in their association factors (such as p43, ARC1, and Trbp111 isolated from various species) [26]; this is because we have not used it in our hybrid approach. The main limitation is that ~38% aaRSs do not contain any of these distinguishable domains. We implemented SVM for the prediction and classification of aaRSs because machine learning-based approaches were essential for the development of prediction tools. We have found that composition of 18 dipeptides (CL, DR, FY, GM, GR, GS, IN, MV, ND, PL, QI, QR, RD, RF, ST, WF, YD, YV) were significantly different in aaRSs and non-aaRSs. The fraction of 14 dipeptides (AY, DP, DW, EN, EV, GH, IG, KM, PW, QW, SG, WD, YA, YV) is different in class-1 and class-2 aaRSs. The SVM module based on hybrid approach^2 ^[Dipeptide composition and PROSITE] achieved higher accuracy in comparison to amino acid composition, dipeptide composition and hybrid approach^1 ^[amino acid composition and PROSITE], both in the prediction of aaRSs and class-specific predictions. It showed that the aaRSs or class-specific aaRSs that failed to be predicted by dipeptide composition could be predicted by hybrid approaches^2 ^containing additional information of PROSITE. These domains contain information about the constant regions inside the aaRSs protein sequences during their development and throughout evolution. Therefore, it is better to use the combined approach for functional analysis where there are unique domains. We implemented all approaches in our web-server *"icaars" *and by default, it uses a hybrid approach^2^. We anticipate that users will be willing to query many sequences at a time and our online server "*icaars*" will take time accordingly. We are in the process of developing stand-alone version to help annotation faster. We hope that an annotation of aaRSs enzyme will be helpful for the designing of new drug-targets and drug-discovery processes.

## Conclusions

To conclude, the present work is an attempt to predict and classify aminoacyl tRNA synthetases. We analysed protein sequences of aaRSs (class-1 and class-2) and non-aaRSs and selected the distinguishable patterns. These were amino acid, dipeptide, hybrid approach^1 ^and hybrid approach^2^. We used these features as a SVM input based machine learning. We were able to model an efficient classifier from hybrid approach^2 ^based information. A server *icaars *has been developed based on the SVM modules obtained.

## Methods

### Datasets

The aaRSs dataset of total 11318 protein sequences was obtained from the ENZYME database release 57.5 of 07-07-2009 [32], which is available through the ExPASy WWW server http://www.expasy.ch/enzyme/. These total 11318 protein sequences of aaRSs, had 4840 class-1 aaRSs and 6478 class-2 aaRSs sequences. We selected non-aaRSs (negative dataset) from the Swiss-Prot database, which were proteins but not tRNA synthetases and removed the sequence similarity from the CD-HIT software [33]. We have further created different non-redundant datasets for aaRSs, aaRS class-1 and aaRSs class-2 by using CD-HIT. The total number of protein sequences in different datasets is given in Table 6. In this study, we have used only 30% non-redundant datasets. This main dataset contains 117, 117, 53 and 65 protein sequences of aaRSs, non-aaRSs, class-1 aaRSs and class-2 aaRSs respectively. Furthermore, we created a realistic dataset of total 117 aaRSs and 1200 non-aaRSs. In addition, we also created independent dataset of aaRSs from 1293 newly added tRNA synthetases in Swiss-Prot database. The 30% non-redundant independent dataset contains 44 aaRSs having 18 sequences of class-1 and 26 sequences of class-2 aaRSs. We have also created independent negative dataset of 450 non-aaRSs.

**Table 6:** Number of protein sequences of aaRSs, class-1 aaRSs and class-2 aaRSs at different redundancy level by using CD-HIT software.

Redundancy	aaRSs	aaRSs (class-1)	aaRSs (class-2)
100%	9889	4346	5547

90%	6487	2939	3551

80%	4998	2305	2695

70%	3686	1750	1938

60%	2369	1157	1214

50%	1330	666	666

40%	669	346	325

30%	117	53	65

### Evaluation method

Firstly, we have used aaRSs as positive dataset and non-aaRSs as negative dataset for the development of the tools for prediction of aaRSs. Proteins sequences of both positive and negative datasets were divided into five parts. Each of these five sets consists of one-fifth of aaRSs and one-fifth of non-aaRSs. For training, testing and evaluating our methods, we have used a five-fold cross-validation technique. In this technique, the training and testing was carried out five times, each time using one distinct set for testing and the remaining four sets for training [34]. Secondly, we used aaRSs class-1 as positive dataset and aaRSs class-2 as negative dataset, repeated above mentioned five-fold cross-validation method for the tools development for discrimination between class-1 and class-2 aaRSs.

### PROSITE and InterProScan

PROSITE is a database of protein families and domains. It is apparent, when studying protein sequences, that during evolution all protein families conserve some portion of protein sequences for efficient function/performance and/or stability of three-dimensional structure, which distinguishes its members from all other unrelated proteins [26]. InterProScan (iprscan) is a Perl-based stand-alone tool that combines different proteins' signature-recognition methods into a single resource [27]. PROSITE database is an integrated part of InterProScan. We applied 4.3 version of InterProScan tool for the PROSITE-based ProfileScan method for the all datasets of aaRSs, aaRSs class-1, aaRSs class-2 and Non-aaRSs. This is a weight matrix-based technique and useful for the detection of diverse protein sequences.

### Amino acid and Dipeptide Composition

The aim of calculating the composition of proteins is to perform the variable length of protein sequences to fixed length feature vectors. This is important and crucial step because SVM machine learning techniques require fixed length patterns. The amino acid composition is the fraction of each amino acid in a protein sequence and provides vector of 20 dimensions. The dipeptide composition was used to encapsulate the global information about each protein sequence, which gives a fixed length pattern of 400 (20 × 20) dimensions of vector. Both amino acids and dipeptide composition was calculated, and used as input to classification between aaRSs and non-aaRSs as well as aaRS Class-1 and aaRSs class-2 by using machine learning of SVM.

### Support Vector Machine (SVM)

In this study, a highly successful machine learning technique termed as a *Support Vector Machine *was used. SVM is based on the structural risk minimization principle of statistics learning theory and SVMs are a set of related supervised learning methods used for classification and regression [35]. SVM allows us to choose a number of parameters and kernels (*e.g*. Linear, polynomial, radial basis function and sigmoidal) or any user-defined kernel. In this study, we implemented SVM^light ^Version 6.01 package [36] of SVM and learning was carried out by using three (linear, polynomial and radial basis function) kernels. SVM takes a set of feature vectors as input, along with their output, which is used for training of model. After training, learned model can be used for prediction of unknown examples [37]. In this work, the SVM training has been carried out by the optimization of various kernel function parameters and the value of the regularization parameter C. Preliminary tests showed that the radial basis function (RBF) kernel gives better results than other kernels. Therefore, in this work, the RBF kernel was used for all the experiments. Total four methods, are used for the SVM-based machine learning and these methods were amino acid composition, dipeptide composition, hybrid approach^1 ^[amino acid composition and PROSITE] and hybrid approach^2 ^[dipeptide composition and PROSITE] based.

### Hybrid approach of SVM and PROSITE

**Figure 8:**
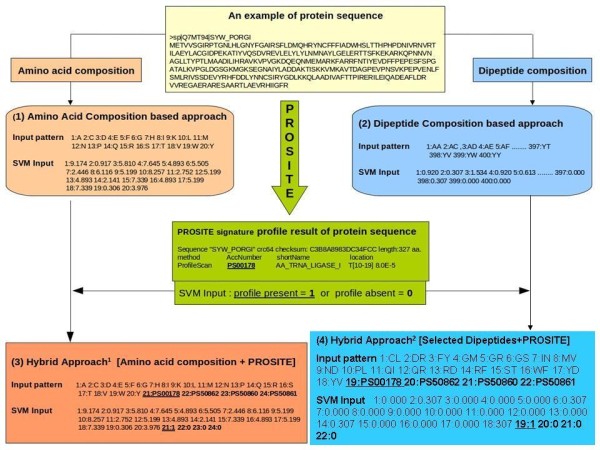
**A flow chart of the SVM input pattern preparation for all five approaches from single example protein sequence**.

It is very beneficial to combine two or more (different) approaches for machine learning techniques. Previously many studies used this approach for the prediction of specific protein families, but in these earlier hybrid-based predictions, at a time one approach was used (e.g. Pfam) but if that did not work then the second approach was utilized [38]. However, in our hybrid approach PROSITE and SVM have been used simultaneously in generating the input patterns for machine learning. This hybrid approach of SVM and PROSITE was the first time used to exploit the benefits of both *de novo *prediction of SVM and PROSITE-based evolutionary information of domains. We analysed that many aaRSs lack any domain by PROSITE. In the prediction between aaRSs and non-aaRSs, four most informative and distinguishable domains were selected from PROSITE by using ProfileScan method of InterProScan tool and prepared hybrid with amino acids and dipeptide composition. We used 1 for the presence and 0 for the absence of domain in particular protein sequence. Total length of input of SVM is increased by four; now there are a total of 24 dimensions of vector for hybrid approach^1 ^[amino acid and PROSITE] and 404 for the all dipeptide composition and PROSITE. In the hybrid approach^2^, we combined 18 selected dipeptides with 4 PROSITE domain features and created total 22 dimensions of vector (figure 8). In the case of prediction between class-1 and class-2 aaRSs total five domains were used: Total 25 vector for hybrid approach^1 ^and 405 for the all dipeptide composition and PROSITE. In the hybrid approach^2^, we combined 14 selected dipeptides with 5 PROSITE domain features and created total 19 dimensions of vector. This hybrid approach contains additional information of the domain-based evolutionary information and SVM-based machine learning technique used this information for the better discrimination between aaRSs and non-aaRSs or class-1 and class-2 aaRSs.

#### BLAST based approach

One of the common practices for predicting the function of a new protein is to perform a similarity search against a database of well-annotated proteins. In this study, we used BLAST for predicting tRNA synthetase proteins using 5-fold cross-validation where four sets of aaRS and non-aaRS proteins were used to create a BLAST database and aaRSs proteins of the corresponding test set were searched against this BLAST database. This process was repeated five times so the BLAST search was performed once for each tRNA synthetase protein. We have calculated performance of BLAST in term of accuracy (percentage coverage); it indicates the correctly predicted proteins from the BLAST search. The number of positive and negative sequence those have not any hit (target) considered as false negative and false positive respectively.

#### Feature Selection method

We have selected most significant dipeptides from all datasets by using WEKA 3.6.0 version [39]. WEKA is a package of java programs for machine learning. We have used attribute evaluator for SVMAttributeEval (parameter -X 1 -Y 0 -Z 0 -P 1.0E-25 -T 1.0E-10 -C 1.0 -N 0) method with ranker (parameter -T -1.7976931348623157E308 -N -1). We have used these selected dipeptide composition with PROSITE domains for the hybrid approaches.

### Evaluation Parameters

The evaluation of performance of method done by calculating the sensitivity, specificity, accuracy and the MCC of the prediction, which were routinely used in these types of studies [40,41]. These parameters can be calculated using equation 1-4,

(1) Sensitivity=[TP/(TP+FN)]×100

(2) Specificity=[TN/(TN+FP)]×100

(3) Accuracy=[(TP+TN)/(TP+TN+FP+FN)]×100

(4) $$MCC=\frac{\left(TP)(TN)-(FP)(FN\right)}{\sqrt{\left[TP+FP][TP+FN][TN+FP][TN+FN\right]}}$$

Where is:

(i) In the case of prediction between aaRSs and non-aaRSs -

TP is correctly predicted positive (aaRSs) proteins

TN is correctly predicted negative (non-aaRSs) proteins

FP is wrongly predicted positive (aaRSs) proteins

FN is wrongly predicted negative (non-aaRSs) proteins.

(ii) In the case of prediction between aaRSs class-1 and aaRSs class-2 -

TP is correctly predicted positive (aaRSs class-1) proteins

TN is correctly predicted negative (aaRSs class-2) proteins

FP is wrongly predicted positive (aaRSs class-1) proteins

FN is wrongly predicted negative (aaRSs class-2) proteins.

The performance of a method is an average of five sub sets, created by five-fold cross validation technique. For the evaluation of any prediction method MCC is considered to the most robust parameter [42]. The MCC value of 1 corresponds to a perfect prediction, whereas 0 corresponds to a completely random prediction. The limitation of all above described parameters that they are threshold-dependent and they require proper optimization for the better performance. We manually optimized all these parameters and selected the one which gives best performance. All the measures described above have a common drawback that their performance depends on threshold selected. A known threshold independent parameter is Receiver Operating Curve (ROC). It is a plot between true positive proportion (TP/TP+FN) and false positive proportion (FP/FP+TN). We have used SPSS package to plot ROC.

## Web-server

We have developed a user-friendly web-server "icaars" for the prediction of aaRSs. This prediction method is freely available from URL http://www.imtech.res.in/raghava/icaars/. It is developed under Solaris envronment on SUN system, using CGI-PERL. This server predicts whether query protein sequence is aaRSs or non-aaRSs. If a protein sequence is predicted as aaRSs then it will further predict whether the protein sequence belongs to class-1 or class-2 aaRSs. All datasets used in this study are available from this server.

## Authors' contributions

BP created datasets and developed the SVM models, backend web server and the front end user interface. GPSR conceived the project, coordinated it and refined the final manuscript drafted by BP. Both authors have read and approved this manuscript.
